# Simultaneous outlet surgery for bladder stones and BPO: a scoping review from EAU endourology - challenging the traditional approach

**DOI:** 10.1007/s00345-026-06369-2

**Published:** 2026-04-06

**Authors:** Enes Dogan, Selim Soytürk, Abdullah Altunhan, Naeem Bhojani, Bhaskar Kumar Somani, Kamran Ahmed, Thomas RW Herrmann, Selcuk Guven

**Affiliations:** 1 Department of Urology, School of Medicine, Necmettin Erbakan University, Konya, Türkiye Turkey; 2 Division of Urology, Université de Montréal, Montréal, PQ Canada; 3 Department of Urology, University Hospital, Southampton, UK; 4 Sheikh Khalifa Medical City, Abu Dhabi, UAE; 5 Khalifa University, Abu Dhabi, UAE; 6 King’s College London, Guy’s and St. Thomas’ NHS Foundation Trust, King’s Health Partners, London, UK; 7 Department of Urology, Spital Thurgau AG, Frauenfeld, Switzerland; 8 Division of Urology, Department of Surgical Sciences, Stellenbosch University, Western Cape, South Africa; 9 Hannover Medical School, Hannover, Germany

**Keywords:** Bladder calculi, Benign Prostatic Obstruction, Cystolithotripsy, Lower Urinary Tract Symptoms, Recurrence

## Abstract

**Objective:**

Management of bladder stones in men with benign prostatic obstruction (BPO) conventionally assumes simultaneous outlet surgery is universally required. This scoping review evaluates evidence comparing simultaneous and staged strategies to establish rationale for selective management.

**Methods:**

The protocol was registered in the Open Science Framework (OSF: https://osf.io/egmz7). Following Preferred Reporting Items for Systematic Reviews and Meta-Analyses extension for Scoping Reviews (PRISMA-ScR) guidelines, PubMed, the Cochrane Library, and Web of Science were systematically searched for studies published up to September 18, 2025, Studies reporting outcomes of stone removal with or without concurrent outlet surgery in patients with bladder stones and BPO were included. Outcomes included stone-free rate (SFR), recurrence, voiding parameters (IPSS, Qmax, PVR), complications, and reintervention.

**Results:**

Fifteen studies (six comparative) met criteria. Both approaches achieved high SFRs (> 90%). Recurrence was lower after combined procedures (≤ 5%) versus isolated stone removal (10–25%), diverging beyond 2–3 years; however, most men receiving stone removal alone did not require subsequent outlet surgery. Combined procedures produced greater LUTS improvement, particularly with prostate volumes > 100 mL or PVR > 90 mL. Early morbidity was higher with simultaneous surgery. No study incorporated urodynamic testing, limiting physiologic interpretation. Key functional domains (ejaculatory function, continence, patient-reported outcomes) and minimally invasive surgical therapies were rarely evaluated.

**Conclusions:**

Available evidence does not support routine simultaneous outlet surgery for all men with bladder stones. The presumed equivalence of stone presence with obstruction, and prostate size with surgical indication, lacks consistent validation. A selective strategy incorporating postoperative re-evaluation, risk stratification, and patient-centered priorities offers a more physiologic, evidence-aligned approach while avoiding overtreatment.

**Supplementary Information:**

The online version contains supplementary material available at 10.1007/s00345-026-06369-2.

## Introduction

Bladder stones and benign prostatic obstruction (BPO) frequently coexist and have traditionally been regarded as pathophysiologically linked conditions. For decades, clinical practice has been shaped by the assumption that the presence of a bladder stone in older men implies clinically significant obstruction and that simultaneous outlet surgery during stone removal is therefore advisable [1–3]. These assumptions arise largely from indirect and heterogeneous evidence, and their universal applicability has been increasingly questioned.

Several factors contribute to this uncertainty. Advances in endoscopic stone management and the widespread use of medical therapy for BPO have expanded therapeutic options [2, 4–6]. In addition, bladder stone formation is no longer viewed as a purely mechanical consequence of obstruction. Infection, biofilm activity, mucosal injury, crystallization, and metabolic influences may also play a role [5–9]. Consequently, a stone does not necessarily confirm functional obstruction, and the relative contribution of the stone, the outlet, or both, can be difficult to determine in individual patients.

Clinical decision-making is therefore complex. While immediate outlet surgery and stone removal are often performed together, staged management with postoperative reassessment is also used in practice. Patient-centred aspects such as continence, ejaculatory function, and quality of life may influence treatment selection, yet these aspects are not always evaluated in a standardized way. Proposed risk indicators, including prostate size and post-void residual volume [10], derive mainly from retrospective cohorts and may not fully reflect functional obstruction. The role of minimally invasive surgical therapies in this setting remains uncertain and should currently be viewed as exploratory rather than established [11, 12].

To address these questions, we conducted a scoping review of studies comparing simultaneous outlet surgery plus stone removal with isolated stone removal, with or without medical therapy, in men with bladder stones and BPO.

## Methods

### Search strategy

This scoping review followed the Preferred Reporting Items for Systematic Reviews and Meta-Analyses extension for Scoping Reviews (PRISMA-ScR) guidelines [13]. A comprehensive search was conducted across three major electronic databases: PubMed, Cochrane Library, and Web of Science, covering all available studies from their inception to September 18, 2025. The search strategy combined controlled vocabulary (MeSH terms) and free-text keywords related to bladder stones and benign prostatic obstruction. The following terms were applied: “Bladder stones,” “Bladder calculi,” “Benign Prostatic Hyperplasia,” “Benign Prostatic Obstruction.” The search was limited to studies published in English. After removal of 18 duplicates, a total of 872 records were screened.(PM:617, CL:80, Wos:193).

### Study selection

Three independent reviewers (E.D, S.S, A.A) screened titles and abstracts, with a fourth reviewer (S.G.) resolving disagreements. A total of 890 studies were evaluated at this stage. Of these, 846 were excluded for not meeting the predefined eligibility criteria. The remaining 26 full-text articles were assessed for eligibility, and 11 were excluded due to being case reports (*n* = 5), editorials (*n* = 4), or review articles (*n* = 2). Finally, 15 studies were included in this scoping review. Studies were deemed eligible if they included adult male patients with concomitant bladder stones and BPO, evaluating either surgical or medical management, and reported clinical outcomes Exclusion criteria comprised studies involving pediatric populations or animal models, as well as those focusing exclusively on bladder stones without BPO or on BPO without concomitant bladder stones.

**Fig. 1:**
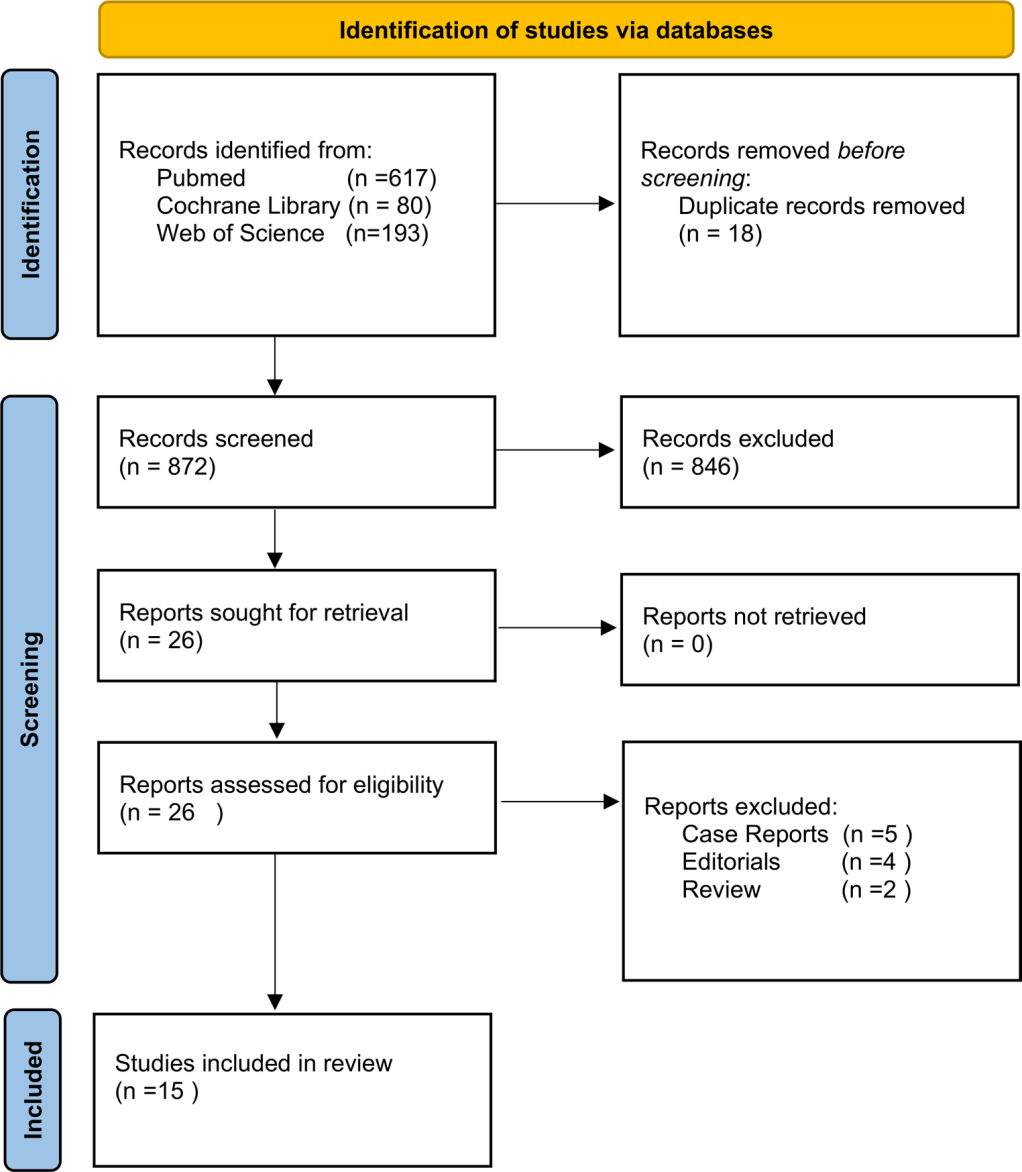
PRISMA-ScR flow diagram of the study selection

A standardized data extraction protocol was applied by two independent reviewers, with discrepancies resolved by a third reviewer. Extracted information included study characteristics such as author, year, design, sample size, mean age, prostate volume, bladder stone features, type of prostate surgery, stone management approach, follow-up duration, and whether any urodynamic assessment was performed. Clinical outcome variables encompassed stone recurrence rate, stone-free rate, symptom scores (IPSS, Qmax, post-void residual urine), and both perioperative and postoperative complications, including those classified according to the Clavien–Dindo system when available. Functional outcomes such as sexual dysfunction, retrograde ejaculation, and continence status were also extracted when reported. Reoperation rates and key findings or limitations noted by the authors were documented. All extracted data were organized into evidence tables to enable structured narrative synthesis. The study selection process, including identification, screening, eligibility assessment, and inclusion of studies, is summarized in Fig. 1 following the PRISMA-ScR flow diagram.

### Study qualification

The following tools were used: Risk of Bias 2 (RoB 2) for randomized trials; Risk Of Bias In Non-randomized Studies of Interventions (ROBINS-I) for nonrandomized studies; and Joanna Briggs Institute (JBI) case-series checklist for single-arm reports [14, 15]. Studies were appraised by domain and mapped to a three-level risk-of-bias scale (low, moderate, high) for cross-design synthesis.

Overall, randomized studies demonstrated low to moderate risk of bias, mainly due to unclear allocation concealment and outcome assessor blinding.(Supplementary Fig. 1) Non-randomized studies showed variable quality, with most rated as moderate risk due to potential selection bias and confounding.(Supplementary Fig. 2) Case series were generally of low methodological quality, mainly due to incomplete inclusion, lack of consecutive enrollment, and unclear reporting of presenting sites.(Supplementary Fig. 3).

## Results

Fifteen studies comprising a total of 1,233 male patients with concomitant bladder stones and BPO were included. Six were comparative, evaluating combined outlet and stone surgery versus isolated stone management or medical therapy, while nine were non-comparative case series (Table 1). The mean age of patients ranged between 38 and 82 years. Reported prostate volumes varied from 26 mL to 106 mL, and the stone burden ranged from single stones smaller than 3 cm to multiple stones larger than 5 cm.

**Table 1:** Summary of Included Studies Comparing Management Approaches for Bladder Stones in Men with Benign Prostatic Obstruction (BPO)

Author, Year	Study Design	Number of Patients (N)	Mean Age (± SD)	Prostate Volume (ml)	Bladder Stone Size(cm)	Prostatectomy Type (TURP, open, HoLEP, etc.)	Stone treatment (simultaneous or method)	Follow-up period (months)	Symptom Scores (IPSS change, Qmax, residual urine)	Stone-Free Rate(%)	Stone Recurrence (%)	Complications (include Clavien-Dindo classification if available)	Reoperation Rate
Yoshida et al., 2015	Retrospective	34	71.9 ± 6.5	57.7 ± 38.7	2.35 ± 1.3	None	TUCL	52.6 ± 30.9	IPSS: 13.5 → 9.7; QoL: 3.8 → 2.4; PVR: 41.4 → 26.1 mL	Majority remained stone-free	17.6	Recurrent UTI (2.9%), urinary retention (5.9%), recurrent stones (17.6%), no renal failure	17.6% (TURP 11.8%, catheterization 5.8%)
O’Connor et al., 2002	Retrospective	23	70.4 (range 38–82)	NR	2.8	None	TUCL	30 (6–96)	IPSS: 18.3 → 9.4; PVR: 354 → 179 mL	NR	17.4	UTI (21.7%), urinary retention (17.4%), chronic renal insufficiency (4.3%), no renal failure	One TURP; 3 repeat stone removals
Nerli et al., 2023	Prospective	37	70.65 ± 5.6	55.49 ± 13.66	4.38 ± 2.92	TURP (14 monopolar, 12 bipolar), HoLEP (11)	5 TUCL, 17 PCCL, 15 OCL	9–48	Qmax 18.65 ± 2.56 mL/s	100	0	Minor complications: wound infection, bleeding, fever; 8.1% transfusion	NR
Sinik et al., 1998	Retrospective	52	63.2 ± 7.1 (52–75)	53.3 ± 7.3	2.1 ± 0.8 × 1.5 ± 0.7	TURP (simultaneous with lithotripsy)	Pneumatic lithotripsy	11.4 (6–18)	NR	100	0	13%: mild hematuria (81%), bleeding, clot retention, urethral/bladder neck/meatal stenosis	NR
Chtourou et al., 2001	Retrospective	120	67.5 (56–85)	35.4 (26–62)	1.85	TURP	Ballistic lithotripsy	9.6 (5–18)	NR	97.5	0	Intra-op: mild hematuria 32%; Post-op: mild bleeding 4, clot retention 1	3 open conversions for large/multiple stones
Savin et al., 2025	Retrospective	63	69 (63–74)	74 (50–106)	2.3	None	TUCL: 26%, 74%PCCL	34 (11–66)	IPSS.NR median Qmax 9 mL/s	100	27	57% had BPO-related complications: hematuria, UTI, retention; all minor (25% Clavien I–II)	22% underwent later BPO surgery (TURP, HoLEP, Aquablation, prostatectomy)
Asci et al., 1999	Retrospective	190	SRA: 55.4; CST: 58.0; TURP: 59.7	NR	2.2 mm (1–3.5)	TURP	TUCL	13 (OMC), 14 (OMC + TURP), 11 (TURP)	Qmax: OMC + TURP 7.2 → 14.3 mL/s; TURP 8.6 → 15.2 mL/s	OMC 94; OMC + TURP 93; TURP N/A	NR	Complications: 21% combined, 13% OMC, 5% TURP (hematuria, urethral stricture, mucosal injuries, TURP syndrome)	Repeat OMC for residual stones in 4 cases
Maresca et al., 2022	Retrospective	103	72 (67–79)	NR	BS any size/number; 40% prior upper tract stones	Groups: no BPO treatment, medical (α-blocker/5ARI), or surgical (TURP)	TUCL (99%), PCCL: (1%)	46 (22–87)	NR	92 after first; 100 ≤ 2 procedures	17 overall; 29 with medical, 18 no BPO, 3% with surgery	No major peri-op complications; minor bleeding 6%, 2 bladder perforations	36% required later BPO treatment (meds, surgery, catheter)
El-Halwagy et al., 2013	Prospective	59	64.6 (50–75)	NR	2.4 (1.5–3.5)	None	ESWL	24	IPSS:21 → 11; Qmax 7.9 → 14.2 mL/s; PVR reduced	96.6	10	Mild hematuria in 5%; dysuria in 7.5%	NR
Ali et al., 2020	Prospective	32	58 (45–70)	67	> 3.5	TURP	OCL	12	IPSS 24 → 8; Qmax 6.5 → 16.8 mL/s	100	0	Complications: mild hematuria 12.5%, wound infection 6%	NR
Philippou et al., 2011	Prospective	64	72 (65–80)	NR	2.7	TURP	TUCL	28	IPSS & Qmax improved significantly in both groups	100 (both groups)	3	NR	34% of cystolithotripsy-only group required later TURP
Mekke et al., 2021	Retrospective	127	CST: 74; SRA: 70	NR	1.8	TURP	TUCL	48	NR	Post-op both 100%	5 with CST vs 19 SRA	Similar complications; TURP ↑ op time (58 vs 33 min) & stay (3.4 vs 1.6 d)	16–18% required re-TURP later
Millán-Rodríguez et al., 2005	Prospective	50	65 (61–69)	NR	< 4	None	ESWL	22	IPSS 17.7 → 9.7; QoL 4 → 1.9	93 after ESWL (77 with single session)	4	No major complications reported; 24% required medical BPO treatment	8% required TURP later (all had high baseline IPSS)
Hasan et al., 2021	Prospective	100	CST: 67,5 SRA 57,5	57–65	< 2.5	TURP	TUCL	20( 12–24)	IPSS, Qmax, PVR improved in both; greater improvement in TURP group	100	Medical group:4, TURP:0	Complications: 4 blood transfusions, 4 bladder perforations (managed conservatively)	30% from medical group required TURP
Caroline Chapelle et al., 2024	Retrospective	179	CST:72 SRA:68	CST:63 SRA:53	< 1 (21%), 1–3 (55%), > 3 (23%)	TURP (60%), GreenLight PVP (14%), Open adenomectomy (14%), TUIP (14%)	TUCL	42	CST patients had higher pre-op PVR (105 vs 30 mL; *p* < 0.001) and better functional success (74.7% vs 55.5%)	100	CST:12 SRA: 39	Early: 51% vs 35%; Late: 26% vs 17%. Major complications rare (Grade ≥ 4 in 1–3%)	CST:14% SRA:44%

BPO: Benign Prostatic Obstruction, BS: Bladder Stone, CST: Concomitant Surgical Treatment, ESWL: Extracorporeal Shock Wave Lithotripsy, HoLEP: Holmium Laser Enucleation of Prostate, IPSS: International Prostate Symptom Score, NR: Not Reported, OCL: Open Cystolithotomy, OMC: Optical Mechanical Cystolithotripsy, PCCL: Percutaneous Cystolitholapaxy, PVP: Photo-vaporization of Prostate, PVR: Post-Void Residual Urine Volume, Qmax: Maximum Urinary Flow Rate, QoL: Quality of Life, SRA: Stone Removal Alone, TUCL: Transurethral Cystolithotripsy/Lapaxy, TUIP: Transurethral Prostate Incision TURP: Transurethral Resection of the Prostate, UTI: Urinary Tract Infection.

## Discussion

This scoping review synthesized the available evidence on the management of bladder stones in men with BPO addressing the ongoing debate on whether concomitant outlet surgery should be routinely performed. Across fifteen studies including 1,233 patients, stone clearance rates were consistently high regardless of the surgical strategy, yet marked variability was observed in recurrence, functional outcomes, and complication profiles. The evidence illustrates a persistent tension between the perceived necessity of treating the underlying outlet obstruction to prevent recurrence and the clinical interest in minimizing perioperative risk and preserving functional outcomes [13]. These findings emphasize that the question is no longer merely technical but physiological and patient-centered requiring a balance between durable relief of obstruction and preservation of sexual and urinary function. Despite the procedural diversity across studies, both isolated and combined approaches achieved comparable short-term efficacy in stone clearance. However, differences emerged in perioperative morbidity, recurrence, and functional recovery, reflecting variations in patient selection and prostate-related characteristics rather than surgical technique alone. The main analytical areas and corresponding evidence limitations identified across studies are summarized in Table 2.The following sections examine these domains in detail, beginning with the outcomes of stone-free rates and procedural efficacy across different surgical strategies.

**Fig. 2:**
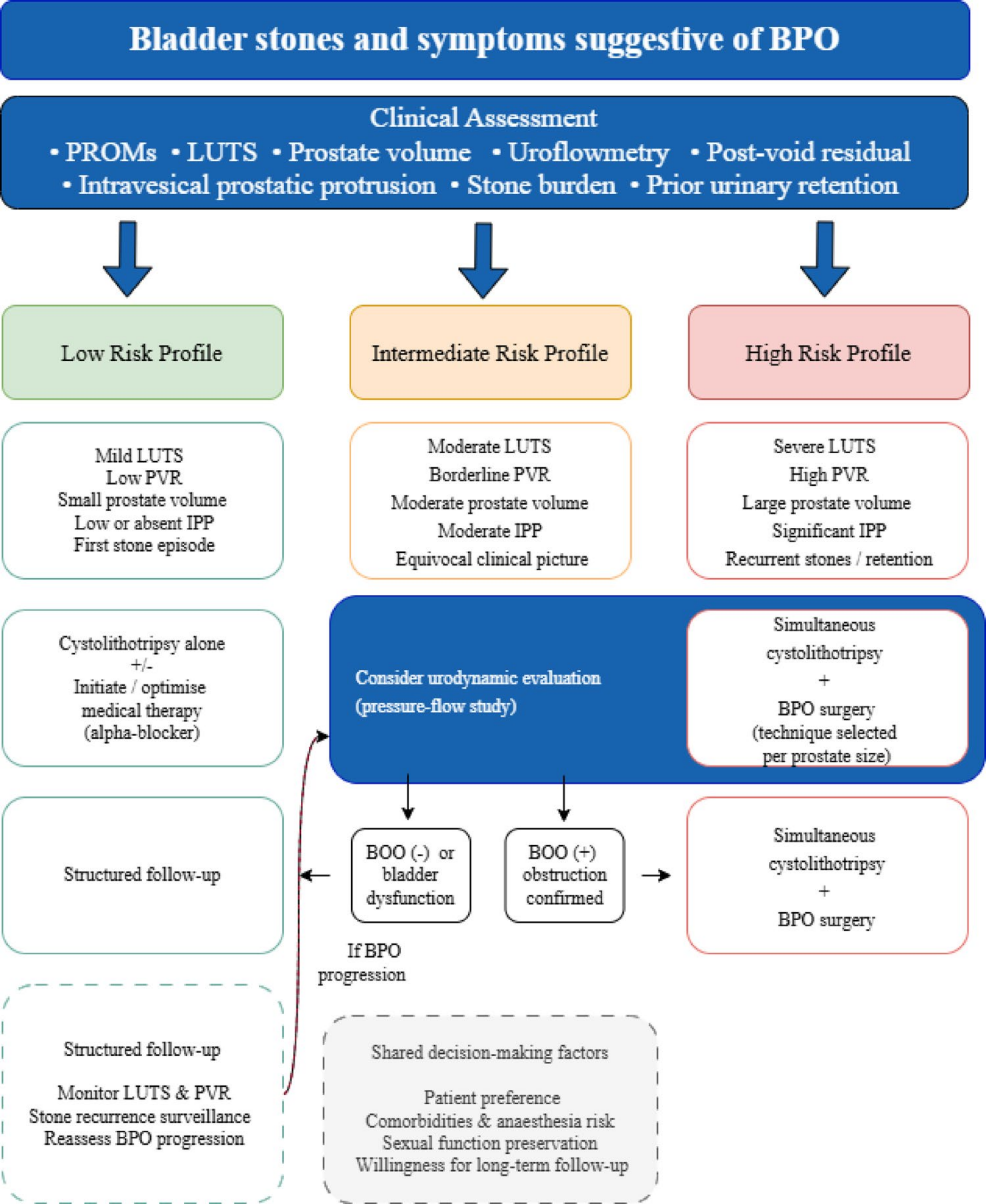
Proposed clinical decision framework for the management of men presenting with bladder stones and benign prostatic obstruction. Abbreviations: IPP = intravesical prostatic protrusion; IPSS = International Prostate Symptom Score; LUTS = lower urinary tract symptoms; PROMS=Patient related outcome measures; PVR = post-void residual; BPO = benign prostatic obstruction; BOO: Bladder outlet obstruction.

## Conclusion

Current evidence does not justify a universal policy of simultaneous outlet surgery for all men presenting with bladder stones and BPO, as this practice carries a substantial risk of overtreatment. While combined procedures offer superior long-term stone-free outcomes and lower recurrence rates, particularly in patients with high-risk features (e.g., prostate volumes > 100 mL or PVRs > 90 mL), they are also associated with higher early morbidity and specific functional complications. Conversely, a staged approach allows for dynamic reassessment of bladder outlet function, and the majority of lower-risk men managed with isolated stone removal do not require subsequent intervention. The most rational, evidence-aligned pathway requires a selective approach based on functional assessment, recurrence risk stratification, and patient-centered counseling. Future research must focus on integrating formal urodynamic evaluation and assessing the role of MIST to refine intervention criteria and minimize unnecessary morbidity.

**Table 2:** Principal analytical themes, their associated factors, and the corresponding evidence limitations identified in studies addressing concomitant bladder stones and benign prostatic obstruction (BPO)

Analytical Themes	Associated Factors	Evidence Limitations
Anatomical vs. Functional Obstruction	Urodynamic findings, PVR, Qmax, IPSS, prostate volume	Volume-based decision-making does not accurately reflect functional obstruction.
Medical Modulation / Downstaging	5-ARI ± α-blocker combination, prostate volume reduction, prevention of AUR	Limited prospective data: outcomes after “watchful waiting” or staged management remain uncertain.
Conventional Outlet Surgery (TURP / AEEP)	TURP, HoLEP, AEEP, and open prostatectomy performed with stone removal	Good long-term data on recurrence prevention, but heterogeneous functional and sexual outcome reporting.
Morbidity vs. Benefit Balance	Ejaculatory dysfunction, incontinence, operative time, hospitalization	QoL and sexual outcomes are rarely reported and lack standardized assessment.
Risk Stratification	High-risk (PVR > 90 mL, volume > 100 mL) vs. lower-risk profiles	No validated or standardized stratification system available.
Large Prostates	Functional heterogeneity in glands ≥ 100 mL; potential for medical regression	Insufficient data on outcomes of medical therapy or staged approaches in this subgroup.
Minimally Invasive Surgical Therapies (MIST)	Role of MIST procedures (e.g., Urolift, Rezūm) in men with bladder stones	No comparative or outcome data available; current discussion remains hypothesis-generating.

Across the reviewed studies, both isolated bladder stone surgery and combined procedures with outlet intervention demonstrated excellent efficacy in achieving stone clearance, with stone-free rates consistently exceeding 90% [16– [18, 21, 22]. This consistency reflects technical maturity across modalities, including transurethral, percutaneous, and optical-mechanical cystolithotripsy, as well as ESWL [7, 16]. The choice between a simultaneous or staged approach appeared to have little impact on immediate clearance, as reported by Millán-Rodríguez, El-Halwagy, and Asci [3, 16, 17], who observed comparable success irrespective of the addition of prostate surgery. While open or endoscopic prostate procedures did not significantly influence short-term stone-free rates, differences in operative time, bleeding risk, and hospitalization were occasionally noted [18, 21]. These variations, however, were not consistently quantified across studies, limiting direct comparison. The predominance of endoscopic approaches in recent years likely accounts for the uniformly high success rates and reduced morbidity, suggesting that technical progress rather than procedural combination has been the major determinant of immediate outcomes [6, 18]. Recent developments in MIST, further expand the treatment spectrum [11, 12]. Although evidence on their concurrent use with cystolithotripsy remains scarce, these approaches may offer a middle ground between conservative management and definitive outlet surgery. Their tissue-sparing characteristics and lower risk of ejaculatory dysfunction position them as attractive alternatives in selected patients, particularly those with small to moderate gland volumes and preserved detrusor function [11, 12]

Recurrence remains the principal argument for performing simultaneous prostate surgery. In this review, recurrence rates ranged from 0–12% following combined procedures and from 3–39% after isolated stone removal [5, 6, 8, 10, 19, 20, 22, 23], with large prostates and elevated post-void residuals identified as key predictors. Studies by Savin, Mekke, and Maresca consistently indicated that untreated outlet obstruction and residual urinary stasis were associated with higher recurrence and reoperation rates [6, 10, 23]. Conversely, several small series demonstrated durable outcomes after isolated stone removal in patients with minimal obstruction or small prostate volumes [19, 20]. These findings suggest that recurrence risk is not universal but patient-dependent, reinforcing the importance of individualized assessment rather than a blanket surgical policy [4, 10]. Stone burden, encompassing both stone size and multiplicity, has been associated with higher recurrence rates in several series, though the underlying mechanism is more likely related to persistent obstruction and urinary stasis than to residual fragments alone [5, 6, 10, 18, 23]., none performed adjusted analyses to determine their independent impact on recurrence. The absence of standardized reporting on stone parameters represents an additional source of heterogeneity and a gap for future research. A staged approach after stone removal allows dynamic reassessment of flow parameters and symptom burden. Postoperative PVR, Qmax, and medical down-staging can help differentiate anatomical enlargement from clinically significant functional obstruction, enabling more precise identification of the subset of men who genuinely benefit from delayed outlet intervention

Functional improvement, measured by IPSS, Qmax, and PVR, was observed in both groups, yet few studies differentiated the source of symptomatic benefit [5, 17, 19, 20, 22]. None of the included studies incorporated formal urodynamic testing, limiting understanding of whether postoperative improvements resulted from reduced obstruction, decreased stone-related irritation, or spontaneous detrusor recovery [3]. Although prostate volume is frequently reported, anatomical enlargement alone does not equate to functional obstruction. Pressure–flow relationships remain the defining criterion, and a substantial proportion of men with reduced flow parameters may not demonstrate obstruction on urodynamic assessment. This distinction is clinically relevant when interpreting treatment effects, as improvement after stone removal may reflect relief of irritative burden rather than outlet resistance itself. The absence of urodynamic data precludes physiologic interpretation of surgical outcomes and underscores a key gap in current evidence [3, 4]. Future studies integrating pressure-flow analyses could clarify which patients genuinely benefit from outlet surgery and which can safely avoid it

Patient expectations represent a crucial but underexplored determinant of treatment strategy. None of the reviewed studies systematically evaluated patient-reported preferences or expectations regarding symptom relief, sexual function, or recovery priorities. In contemporary urological practice, such preferences often guide the decision to include or omit outlet surgery, particularly when long-term recurrence prevention must be weighed against the potential for ejaculatory or continence-related complications [4, 11, 12]. Integrating shared decision-making frameworks and validated patient-reported outcome measures would allow a more holistic approach to managing men with bladder stones and coexisting BPH [4]

Subgroup analyses demonstrated that prostate volume and post-void residual urine were the strongest predictors for subsequent outlet surgery [6, 10, 23]. Savin et al. identified prostate volume > 100 mL and PVR > 93 mL as independent risk factors for delayed BPH surgery [10], while Mekke et al. and Maresca et al. reported similar trends in recurrence and reoperation among men with large glands managed conservatively [6, 23]. However, threshold definitions for “large” prostates varied across studies, and stratified outcome analyses remain limited. The evidence collectively supports considering prostate size and functional capacity, rather than the presence of stones alone, when deciding on outlet intervention [4, 6, 10]. Although larger prostate size and elevated residual urine volumes are recognized prognostic markers for long-term progression, these parameters describe risk rather than immediate surgical necessity. Without demonstration of functional obstruction, applying volume-based thresholds uniformly may lead to unnecessary intervention. Beyond prostate volume and PVR, intravesical prostatic protrusion (IPP) has been proposed as a non-invasive marker of obstruction severity relevant to surgical decision-making. A meta-analysis confirmed that IPP reliably predicts BOO and outperforms prostate volume alone [26], and men with IPP greater than 10 mm have been shown to exhibit higher BOOI, lower Qmax, and more frequent acute urinary retention [27]. Kim et al. identified IPP as an independent risk factor for bladder stone formation in BPH patients [28], and higher IPP grades have been linked to increased rates of stones, infections, and need for surgery [29]. Taken together, these findings suggest that large IPP may identify a subgroup of men in whom simultaneous prostate intervention is more likely to be necessary, as the degree of protrusion reflects both the severity of functional obstruction and the risk of stone recurrence. However, none of the studies included in our review evaluated IPP, and direct evidence supporting its use as a criterion for concurrent surgery in the bladder stone setting is still lacking

Perioperative complications were generally low across studies, reflecting advancements in endoscopic technology and surgical experience. However, combined procedures inherently carried a higher risk of transient bleeding, infection, or retention [2, 17, 18]. Functional and late complications, including urinary incontinence, urethral or bladder neck stricture, and sexual dysfunction, were rarely evaluated in detail. Only Chapelle et al. provided data on incontinence (15% vs. 6%) and sexual dysfunction (1–3%), and none of the studies reported standardized IIEF or MSHQ-EjD scores [8]. Higher early complication rates reported after combined procedures may partly reflect the biological environment in which surgery is performed. Bladder stones frequently harbor bacterial biofilm even in culture-negative urine, and manipulation of prostatic tissue in this setting may increase susceptibility to infectious or inflammatory postoperative events. The evolution of tissue-sparing MIST techniques, such as the prostatic urethral lift and water vapor thermal therapy, introduces a paradigm shift emphasizing functional preservation. PVP and aquablation, although less morbid than conventional resection, are ablative procedures and do not share the same tissue-preserving profile. While data on their concurrent use with cystolithotripsy are lacking, these methods may mitigate risks traditionally associated with transurethral resection or enucleation, particularly retrograde ejaculation and ejaculatory dysfunction. This transition aligns with the broader movement in urology toward minimally invasive, patient-centered surgery [4, 11, 12]. Although evidence remains limited, minimally invasive surgical therapies may represent an intermediate option for selected patients in whom preservation of ejaculatory function, shorter recovery, or reduced perioperative morbidity is prioritized. Their potential role in staged management warrants further investigation

Most available studies were retrospective, single-center analyses with limited follow-up and heterogeneous inclusion criteria. Standardized outcome measures for recurrence, complications, and functional parameters were inconsistently applied, and none of the studies reported formal cost analyses or quality-of-life outcomes. The lack of randomized controlled trials and the absence of urodynamic and patient-reported data restrict the ability to draw definitive conclusions regarding the optimal strategy. Given the functional trade-offs associated with outlet surgery, including ejaculatory dysfunction, transient incontinence, and longer recovery, patient expectations may materially influence treatment selection. The absence of validated patient-reported measures in available studies limits the ability to assess outcomes according to patient priorities, which frequently extend beyond recurrence risk alone

The current evidence suggests that the decision to perform outlet surgery should not be routine but selective, integrating anatomical, functional, and patient-centered factors. Combined procedures appear beneficial in patients with large prostates, high post-void residuals, or proven obstruction, whereas isolated stone removal may be appropriate for those with small glands, mild symptoms, or strong preference for functional preservation. Future prospective studies should incorporate urodynamic testing, standardized reporting of sexual and urinary outcomes, and formal assessment of patient expectations to develop an individualized decision framework balancing efficacy, safety, and quality of life

The current evidence suggests that the decision to perform outlet surgery should not be routine but selective, integrating anatomical, functional, and patient-centered factors. Combined procedures appear beneficial in patients with large prostates, high post-void residuals, or proven obstruction, whereas isolated stone removal may be appropriate for those with small glands, mild symptoms, or strong preference for functional preservation. Future prospective studies should incorporate urodynamic testing, standardized reporting of sexual and urinary outcomes, and formal assessment of patient expectations to develop an individualized decision framework balancing efficacy, safety, and quality of life

Based on the findings of this scoping review, we propose a conceptual clinical decision framework for men presenting with bladder stones and symptoms suggestive of BPO (Fig. 2). The framework stratifies patients into low, intermediate, and high risk profiles for persistent obstruction based on PROMs, LUTS severity, PVR, prostate volume, IPP, and stone recurrence history. In intermediate risk patients with an equivocal clinical picture, and in selected high risk cases, urodynamic evaluation may help guide the decision between simultaneous surgery and cystolithotripsy alone. Specific thresholds for each parameter remain to be established through prospective comparative studies

### Intervention techniques

Stone management techniques included transurethral, percutaneous, and optical-mechanical cystolithotripsy, extracorporeal shock wave lithotripsy (ESWL), and open vesicolithotomy. Concomitant prostate surgery consisted of transurethral resection of the prostate (TURP), open simple prostatectomy, photoselective vaporization of the prostate (PVP), aquablation, and holmium laser enucleation of the prostate (HoLEP). PVP and aquablation, were used in a small number of studies. TURP remained the most frequently performed outlet procedure (10 studies), followed by HoLEP and open prostatectomy in two studies each, and PVP in one study. Follow-up periods ranged from 5 to 96 months. Stone removal was most often endoscopic; several studies performed simultaneous stone and prostate surgery in the same session, whereas others described staged or nonsynchronous approaches.

### Patient expectations

None of the included studies systematically assessed patient expectations or preferences regarding surgical strategy. While several reports mentioned symptomatic relief or satisfaction in descriptive terms, no validated patient-reported outcome measures were used to capture expectations for functional preservation, recovery time, or postoperative quality of life.

### Stone-free rates

All included studies reported stone-free rates exceeding 90% in both simultaneous and staged procedures. Millán-Rodríguez et al. achieved a 93.2% clearance rate after ESWL, with 77% of patients requiring one session [3]. El-Halwagy et al. reported 96.6% success within six weeks [16]. Asci et al. reported rates of 94% following optical-mechanical cystolithotripsy (OMC) and 93% after OMC + TURP, while Chtourou et al. documented complete fragmentation in 97.5% of 117 patients [17, 18]. Eleven additional studies reported 100% clearance [5, 6, 8, 19].

### Stone recurrence

Thirteen studies provided data on stone recurrence. The recurrence rate ranged from 0 to 12% in groups undergoing concurrent prostate surgery and from 3.1 to 39% among those treated without outlet surgery. Yoshida et al. reported recurrence in 17.6% of patients, O’Connor et al. in 17.4%, and Savin et al. in 27% [10, 19, 20]. No recurrence was observed by Ali et al. during 12 months of follow-up [21]. Philippou et al. found comparable recurrence rates (3.1%) in both cystolithotripsy + TURP and cystolithotripsy + medical (tamsulosin and finasteride) for LUTS arms [5]. Mekke et al. reported 5% recurrence in the TURP group and 19% in the non-TURP group [6]. Millán-Rodríguez et al. observed 4%, Hasan et al. 0% following TURP + cystolitholapaxy and 6% with cystolitholapaxy + medical therapy, while Maresca et al. documented 3% after surgical BPO treatment, 29% after medical therapy, and 18% without BPO treatment [3, 22, 23]. Chapelle et al. found recurrence in 39% of patients treated with stone removal alone and 12% following concomitant surgery [8]. Four studies reported 0% recurrence, and two did not provide recurrence data. Data regarding stone size and number were heterogeneously reported across the included studies. In general, series involving multiple or large bladder stones (> 3 cm) tended to show higher recurrence and reoperation rates, particularly when treated without concomitant outlet surgery. However, formal stratified statistical comparisons were lacking, limiting quantitative interpretation of these effects. While combined procedures reduce recurrence risk, several comparative studies indicate that the majority of men treated with isolated stone removal do not subsequently require outlet surgery.

### Reoperation

Reoperation rates were reported in 11 studies, ranging from 0 to 21% following concurrent prostate surgery and from 4.3 to 44% without concurrent surgery. Yoshida et al. reported invasive intervention in 17.6% of patients, including TURP for recurrent bladder stones in four patients and catheterization for urinary retention in two. O’Connor et al. reported that one patient (4.3%) required TURP following recurrent stone formation. Chtourou et al. reported no reoperation for either recurrent stones or BPO during a mean follow-up of 9.6 months [18–20]. Savin et al. reported that 22% eventually underwent BPO surgery during follow-up, while stone recurrence (27%) and urinary retention requiring hospitalization (14%) were reported as separate secondary outcomes [10]. Asci et al. observed 6.6% reoperation in the OMC + TURP group, 6.3% in OMC alone, and 0% in TURP alone [17]. Maresca et al. reported reoperation for stones in 18% of patients without BPO treatment, 29% with medical therapy, and 3% after BPO surgery; overall, 35% required later unplanned BPO intervention [23]. Philippou et al. observed that 34.3% of medically managed patients subsequently underwent TURP, while 3.1% of surgical patients required re-TURP [5]. Mekke et al. reported repeat cystolithotripsy in 5% of TURP patients and 19% without TURP, repeat TURP in 16% and 18%, with overall reoperation rates of 21% and 37%, respectively [6]. Millán-Rodríguez et al. noted 8% subsequent TURP, Hasan et al. 0% in TURP and 30% in medical groups, and Chapelle et al. 14% with concomitant surgery versus 44% after stone removal alone [3, 8, 25].

### Functional outcomes

Twelve studies reported lower urinary tract symptom parameters. In simultaneous surgery groups, O’Connor et al. described improvement in IPSS from 18.3 to 9.4 and PVR from 354 to 179 mL; Ali et al. observed IPSS reduction from 24 to 8 and Qmax increase from 6.5 to 16.8 mL/s; and Nerli et al. reported Qmax improvement from 2.6 to 18.7 mL/s [20, 21, 24]. In studies without concurrent outlet surgery, Yoshida et al. reported a decrease in IPSS from 13.5 to 9.7, PVR from 41.4 to 26.1 mL, and QoL from 3.8 to 2.4; El-Halwagy et al. found IPSS decline from 21 to 11 and Qmax rise from 7.9 to 14.2 mL/s; Millán-Rodríguez et al. observed IPSS reduction from 17.7 to 9.7 and QoL improvement from 4.0 to 1.9 [3, 16, 19]. In comparative analyses, Hasan et al. recorded IPSS improvement from 20 to 9.0 after TURP and from 19.5 to 13.2 with medical therapy, while Asci et al. found Qmax increase from 7.2 to 14.3 mL/s following OMC + TURP [17, 22]. Studies show significant symptom and urinary flow improvements, especially in patients having outlet surgery with concurrent bladder stone management.

### Urodynamic evaluation

None of the included studies incorporated formal urodynamic testing. Although several reports presented post-void residual and uroflowmetry data, parameters such as detrusor pressure, bladder compliance, or contractility were not assessed.

### Large prostates

Several studies included subgroup analyses or reported findings related to prostate volume. Savin et al. identified prostate volume > 100 mL as a significant predictor for subsequent outlet surgery (HR 3.47; 95% CI 1.14–10.52; *p* = 0.03), together with preoperative post-void residual > 93 mL [10]. Mekke et al. and Maresca et al. also noted that men with larger prostates experienced higher rates of stone recurrence and reoperation, particularly when treated without concurrent outlet surgery [6, 23]. In other series, prostate volume ranged between 26 and 106 mL, but stratified analyses for large glands were not performed. Overall, data specific to prostates ≥ 100 mL remained limited and heterogeneous across studies.

### Complications

Among patients undergoing simultaneous prostate surgery, Nerli et al. reported minor complications in 8.1% of cases (bleeding, fever, wound infection), and no major events [24]. Sinik et al. observed minor complications in 13% of patients, primarily mild hematuria and urethral or meatal stenosis [25]. Chtourou et al. reported mild hematuria in 32%, postoperative bleeding in 3.3%, and clot retention in 0.8%, without major complications or mortality. Ali et al. found minor complications in 6.9% (Clavien–Dindo I–II) and no Grade ≥ III events, transfusions, or deaths. In the bicentric study by Chapelle et al., early complications occurred in 51% of patients with concomitant surgery and 35% after stone removal alone (*p* = 0.168), while late complications occurred in 26% and 17% (*p* = 0.229) [8]. Urinary incontinence was reported in 15% versus 6%, major complications in 7% versus 3%, and mortality in 2% versus 0%, respectively.

In studies without concomitant outlet surgery, Yoshida et al. reported an overall complication rate of 23.5%, including recurrent stones (17.6%), urinary retention (5.9%), and urinary tract infection (2.9%) [19]. O’Connor et al. documented urinary tract infection in 21.7%, retention in 17.4%, recurrent stones in 17.4%, and renal insufficiency in 4.3%. Savin et al. reported early minor complications in 25% and no major events [10, 20]. El-Halwagy et al. recorded clinically significant complications in 3.4% of patients, all minor, and Millán-Rodríguez et al. reported no perioperative events, with subsequent recurrent stones in 4% and TURP in 8% [3, 16]. 

In comparative series, Asci et al. reported complication rates of 21% for combined OMC + TURP, 13% for OMC alone, and 5% for TURP alone [17]. Maresca et al. found total complication rates of 29% for stone surgery alone, 37% for medical therapy, and 32% for surgical BPO management, with acute urinary retention (17.5%) as the most frequent event [23]. Philippou et al. observed overall complication rates of 15.6% for cystolithotripsy + TURP and 34.3% for cystolithotripsy + medical therapy (tamsulosin and finasteride) for LUTS, with no major complications [5]. Mekke et al. reported 15.8% in the TURP group and 22.5% in the non-TURP group, with one major event (2.6%) in the TURP cohort [6]. Hasan et al. recorded rates of 12% after TURP and 4% with medical therapy, with no major complications or deaths [22]. 

Functional and late complications were rarely evaluated across studies. Only Chapelle et al. provided specific data on urinary incontinence (15% vs. 6%), urethral stricture (4–7%), and sexual dysfunction (1–3%) in patients with and without concomitant surgery, though no standardized measures such as IIEF or MSHQ-EjD were applied [8]. Other studies did not report on retrograde ejaculation, erectile function, or bladder neck contracture. Overall, data on sexual or quality-of-life–related adverse events were insufficient, as most studies focused on perioperative safety and stone-related outcomes.

Predictive analyses identified larger prostate volume and high post-void residual urine as the strongest risk factors for later outlet surgery. Savin et al. found that prostate volume > 100 mL (HR 3.47; 95% CI 1.14–10.52; *p* = 0.03) and preoperative PVR > 93 mL (HR 3.82; 95% CI 1.07–13.67; *p* = 0.04) independently predicted subsequent BPO surgery [10]. Millán-Rodríguez et al. identified baseline IPSS as a prognostic factor for later prostate surgery (*p* = 0.042) [3]. 

## Supplementary Information

Below is the link to the electronic supplementary material.

Supplementary Fig .(DOCX 5072 KB)

## Data Availability

No datasets were generated or analysed during the current study.
