# Supplementary material for: Simultaneous outlet surgery for bladder stones and BPO: a scoping review from EAU endourology - challenging the traditional approach
Source: World J Urol. 2026 Apr 6;44(1):279. doi: 10.1007/s00345-026-06369-2 (PMC13053587; doi:10.1007/s00345-026-06369-2)
Supplement: Supplementary file 1 — Supplementary Fig .(DOCX 5072 KB) [file 345_2026_6369_MOESM1_ESM.docx]

Supplementary Figure 1: Risk of Bias 2 (RoB 2) for randomized trials


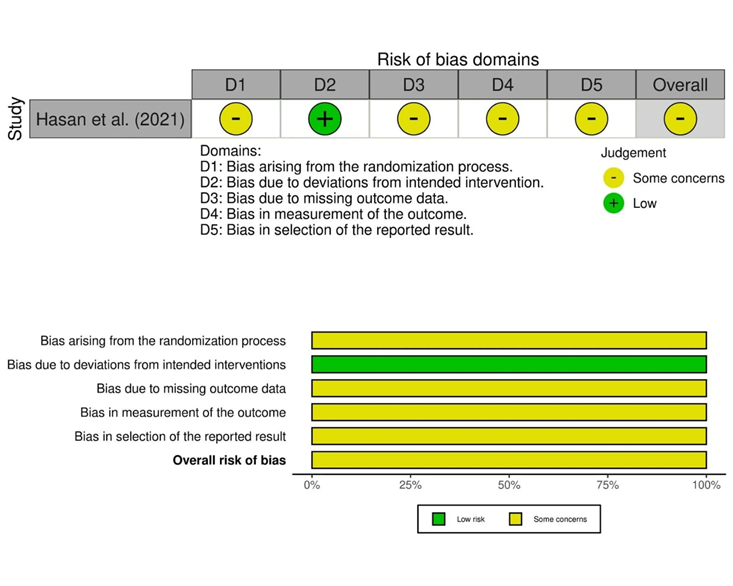


Supplementary Figure 2: Risk of bias assessment (ROBINS-I) for non-randomized studies.
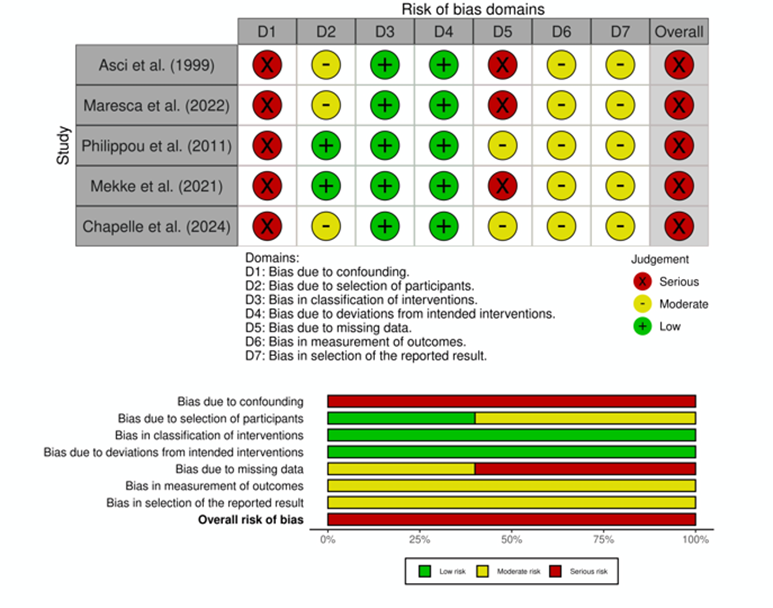


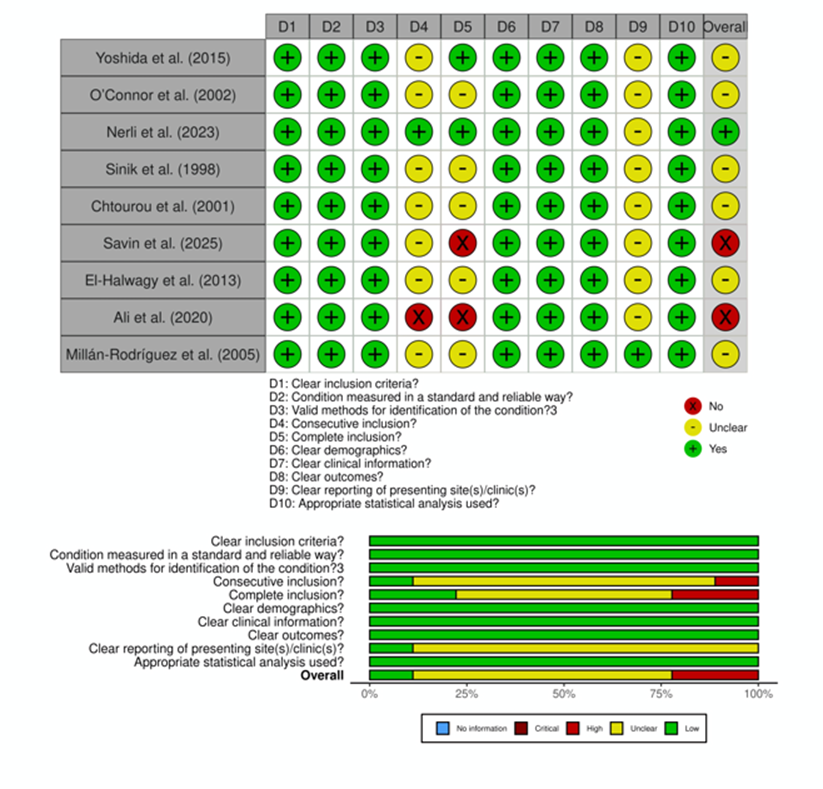
Supplementary Figure 3: JBI quality appraisal for case series.
